# Emotional abuse of girls in Swaziland: prevalence, perpetrators, risk and protective factors and health outcomes

**DOI:** 10.7189/jogh.07.010410

**Published:** 2017-06

**Authors:** Franziska Meinck, Deborah Fry, Choice Ginindza, Kerri Wazny, Aldo Elizalde, Thees F Spreckelsen, M Catherine Maternowska, Michael P Dunne

**Affiliations:** 1 University of Oxford, Oxford, England, UK; 2 OPTENTIA, School of Behavioural Sciences, North–West University, Vanderbijlpark, South Africa; 3 Moray House School of Education, University of Edinburgh, Edinburgh, Scotland, UK; 4 Swaziland Central Statistical Office, Mbabane, Swaziland; 5 Institute of Health and Wellbeing, University of Glasgow, Glasgow, Scotland, UK; 6 UNICEF Office of Research – Innocenti, Florence, Italy; 7 School of Public Health and Social Work, Queensland University of Technology, Brisbane, Australia

## Abstract

**Background:**

Research on emotional child abuse in sub–Saharan Africa is scarce. Few studies thus far have examined prevalence, risk and protective factors for emotional child abuse or the associations between emotional abuse and girls’ health.

**Methods:**

A nationally representative two–stage, cluster–sampled, household survey of females aged 13–24 years (n = 1244) on childhood abuse victimisation was conducted. Participants completed interviewer–assisted questionnaires. Associations between emotional abuse and putative risk, and protective factors and health outcomes were analyzed using separate logistic regression models accounting for sampling design. Marginal effects of cumulative risk factors for emotional abuse victimisation were examined.

**Results:**

Lifetime prevalence of emotional abuse was 28.5% with 58.3% of these girls reporting many abusive incidents. The most common perpetrators were female (27.8%) and male (16.7%) relatives and, more rarely, biological parents. Risk factors associated with emotional abuse were frequent caregiver changes (odds ratio (OR) 1.42, 95% confidence interval (CI) 1.03–1.970, poverty (OR 1.51, 95% CI 1.12–2.03), and physical abuse (OR 1.98, 95% CI 1.45–2.71) and sexual abuse (OR 2.22, 95% CI 1.57–3.10) victimisation. Being close to one’s mother was a protective factor (OR 0.88, 95% CI 0.80–0.97). Risk for emotional abuse increased from 13% with no risk factors present to 58.4% –with all four risk factors present. Health outcomes associated with emotional child abuse were suicidal ideation (OR 1.85, 95% CI 1.30–2.63) and feeling depressed (OR 1.89, 95% CI 1.31–2.71).

**Conclusions:**

Girls in Swaziland experience high levels of emotional abuse victimisation. Emotional abuse is associated with economic disadvantage, family factors, other types of abuse victimisation and poor mental health. Therefore, a holistic approach to prevention is needed, incorporating poverty reduction and programmes to improve parent–child relationships, reduce the use of harsh criticism, and change parenting social norms.

Worldwide, millions of children are victims of abuse and neglect [1], with children in the sub–Saharan African region suffering from particularly high rates of abuse [2,3]. Child maltreatment in the region is associated with a large range of negative outcomes including substance use [4], mental health problems [5], re–victimisation [6], and HIV–risk behavior [7].

A recent systematic review of risk factors for child abuse victimisation in Africa found a growing body of evidence on factors associated mainly with physical and sexual abuse victimisation [8]. Factors pertaining to emotional child abuse, however, are understudied. In fact, across the sub–Saharan region, only a few quantitative studies have investigated determinants or consequences of emotional child abuse, mostly in conjunction with other adverse childhood experiences [5,9–11]. Available research on emotional abuse alone presents a range of findings depending on the measurements used: living or having lived with a step–father [12], witnessing domestic violence [13], having a caregiver who is ill with AIDS or being AIDS–orphaned [7,11], living with someone who is chronically ill, poverty [14], poor family functioning [15] and poor caregiver mental health [13,15]. Countries across sub–Saharan Africa are culturally and socioeconomically diverse with differing services available to children and abuse victims. Therefore, country–specific research on prevalence and factors associated with emotional child abuse is important for the design of prevention and care policies and programs.

In Swaziland, research on emotional abuse has mostly focused on abuse perpetrated by teachers [16] and the use of humiliating punishment [17]. Some studies have identified drivers of physical and emotional violence against children in Swaziland such as poverty [17,18], orphanhood and moving to live with a different caregiver [18]. However, none of these studies used a nationally representative sample of children to investigate factors associated with emotional child abuse.

The current study had four aims: 1) to estimate the prevalence of childhood emotional abuse and frequency of victimisation, along with the most common perpetrators of emotional abuse among girls in Swaziland; 2) to investigate potential risk and protective factors for emotional abuse 3) to examine whether there is a cumulative risk for emotional abuse when more than one risk factor is present; and 4) to identify associations between emotional abuse and health outcomes.

## METHODS

The analyses presented below are part of the Violence Against Girls Study in Swaziland. The Government of Swaziland had the responsibility for the overall study design and management of the national survey with technical leadership provided by the Central Statistical Office in collaboration with UNICEF Swaziland and the United States Center for Disease Control and Prevention (CDC). The study’s overarching aim was to describe epidemiological patterns of sexual violence, identify risk factors, assess knowledge and utilization of services available to victims of sexual violence and to improve awareness of sexual violence against girls [19]. It is to date the only nationally representative survey of girls in Swaziland.

### Procedure

From May 2007 to June 2007, a nationally representative household survey of 13–24 year–old females was conducted using a two–stage, cluster survey design. In the first stage, 40 enumerator areas were selected. In the second stage, a systematic sample of 48 households in each enumerator area with a random start was selected. The sampling frame was provided by the Central Statistics Office of Swaziland based on the 1997 population census. A total of 1900 households were visited, of which 68% (1292) had an eligible female (aged 13–24 years). Overall response rate was 96.3% with 1.1% (n = 14) refusals and 2.6% (n = 34) unavailability [19], which is comparable to other studies in the region [20], and resulted in data from 1244 individuals. One eligible participant was interviewed per household. Where more than one eligible girl was resident, a single participant was randomly selected using the Kish Method [20]. The interview schedule was developed using standardised questionnaires with the help of local informants and were pre–tested through piloting. Interviews were carried out in SiSwati and no incentives were provided. More information on sampling and methodology are available from previous publications on Violence Against Girls in Swaziland [21–23].

Ethical approval was granted by the CDC’s Institutional Review Board, and ethics and safety guidelines for studies on violence against women were followed [24,25]. Voluntary consent was obtained from all participants and their head of household. Questionnaires were completed with the help of female interviewers who had received extensive training on privacy, confidentiality and talking about sensitive themes. Participants could stop the interview at any time or skip questions and were given a list of organisations that provide services for women and children.

### Measures

*Emotional abuse* was measured using one item: “When you were growing up, did any adults scare you or make you feel really bad because they called you names, said mean things to you, or said they didn’t want you?” Any emotional abuse that was carried out by an adult such as a biological parent, another relative, a partner, teacher, community or church leader when the participant was aged <18 was included in this analysis.

*Physical abuse* was measured using one item on being kicked, bitten, slapped, hit with a fist or an item, or threatened with a weapon. Contact *sexual abuse* was measured using five items on forced sexual intercourse, coerced sexual intercourse, attempted forced intercourse, and forced sexual touching. The participant had to be <18 years old when the first such incident occurred.

*Abuse frequency* was measured for each perpetrator with a response code of “never; once; few; and many”.

*Orphanhood* was defined as having lost one or both parents during childhood [26]. Participants were also asked how *close to their biological mother or father* they felt.

*Overcrowding* was measured asking for the largest number of people who lived in the home at any point in time and defined as >5. *Frequent caregiver changes* were measured using one item establishing how many different families the participant had lived with in their lifetime and defined as having moved family >3. *Poverty* was measured using the proxy food insufficiency which was defined as going hungry often or sometimes. *Community trust* was measured using an item on trusting people in the neighborhood/community/village. *School trust* was measured using one item establishing how trusting the child was of teachers and school administrators.

*Mental health and health risk behaviors* were not comprehensively measured in this interview, although indicators of possible mental health problems included one item on *depression* (“have you ever felt depressed?”), two items on *suicidal ideation* (“have you ever thought about suicide and attempted suicide?”), one item on *smoking* cigarettes, and ever drinking *alcohol,* ever having had a *sexually transmitted disease,* and being *HIV–positive*.

Other socio–demographic information was also collected: the *importance of religion*, *faith, highest level of education, marital status,* whether they lived in *urban/peri–urban or rural locations*, and their *age*.

Most of the items used in this survey have been used successfully in multiple Violence Against Children (VAC) studies [23,27,28].

### Analysis

**Figure 1:**
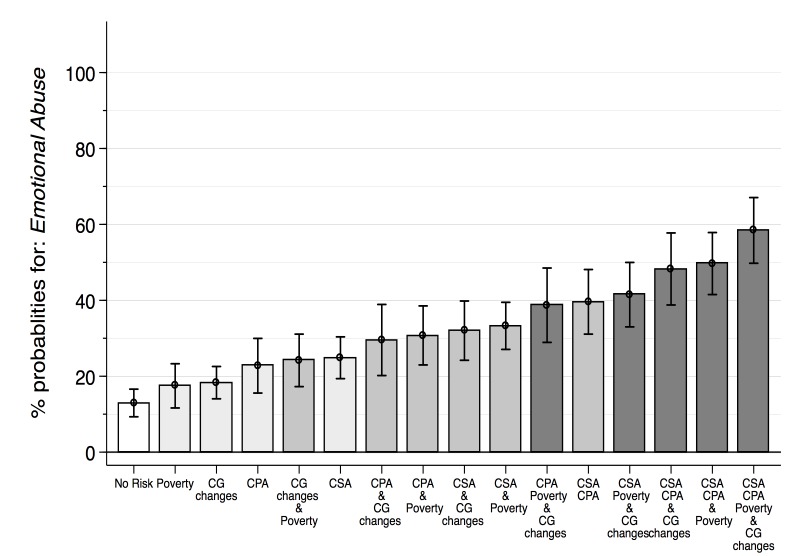
Predicted cumulative risk for emotional abuse victimisation among girls in Swaziland, 2007. Data source: VACS Swaziland, n=1224.

Four analyses steps were conducted using Stata 13 (StataCorp, College Station, Texas, USA), each accounting for the clustered sampling design. First, descriptive statistics for emotional abuse, risk factors, health outcomes and covariates were obtained (**Table 1**). Second, bivariate regressions were used to investigate associations among emotional abuse, hypothesized risk factors and health indicators (**Table 2**). Third, all significant risk factors (at *P* < 0.05) obtained from the bivariate regression were included in two multivariate models with controls for age, urban/rural location and faith. This resulted in five regression models: risk factors for emotional abuse (**Table 3**, Models 1, 2 and 3) and health outcomes (**Table 4**, Models 1 and 2) showing model selection through backward elimination of non–significant risk factors until all remaining factors were associated with emotional abuse (*P* < 0.05) [29]. The first risk factor model includes all significant factors (*P* < 0.05) from the bivariate regressions (Model 1). The second includes all factors significant at *P* < 0.1 and the third includes all factors significant at *P* < 0.05. For the health outcome models, the first includes all risk factors significant at *P* > 0.1. The second includes all factors significant at *P* < 0.05. Finally, cumulative risk of associated factors was tested. For all risk factor variables and covariates that were included in **Table 3** Model 3, marginal effects for emotional abuse – based on the logistic regression model – were calculated with all covariates held at their mean value (average marginal effect). This determined how the predicted probability of the outcome changes for different risk factors (and combinations of risk factors; see **Figure 1**).

**Table 1:** Socio–demographic characteristics of the sample of girls in Swaziland, 2007 (n = 1224)*

Characteristics	Percentage (n)	Confidence Interval (95%)
Child emotional abuse	28.5% (355)	25.8–31.4%
Child sexual abuse	49.3% (613)	46.2–52.5%
Child physical abuse	25.6% (319)	22.5–27.8%
Orphanhood	43.7% (544)	40.6–47.9%
Paternal orphanhood	33.0% (411)	30.4–36.4%
Maternal orphanhood	10.8% (134)	9.0–12.9%
Community trust:		
-Strongly disagree	13.7% (170)	11.7–16.1%
-Disagree	39.6% (493)	36.6–42.7%
-Neither agree nor disagree	18.8% (234)	16.4–21.5%
-Agree	22.0% (274)	19.6–24.6%
-Strongly agree	5.7% (71)	4.5–7.4%
School trust:		
-Not at all trusting	9.0% (112)	7.3–11.0%
-Somewhat trusting	31.1% (387)	28.2–34.1%
-Very trusting	59.9% (745)	56.8–62.9%
Religious practice:		
-Not important	7.2% (90)	5.8–9.0%
-A little important	5.4% (67)	4.1–7.0%
-Somewhat important	16.0% (199)	13.8–18.5%
-Very important	71.4% (888)	68.5–74.1%
Poverty	58.5% (728)	55.5–61.4%
Overcrowding	87.8% (1092)	85.8–89.5%
Close to mother	75.9% (944)	73.2–78.9%
Close to father	46.6% (580)	43.5–49.7%
Frequent caregiver changes	26.1% (325)	23.5–28.9%
Age (years)	17.9 (SE 0.10)	17.8–18.1
Relationship status:		
-Married	9.7% (120)	8.1–11.7%
-Living with partner, not married	3.4% (42)	2.5–4.4%
-Not living with partner	86.8% (1079)	84.7–88.7%
Urban or peri–urban area	15.3% (190)	14.5–16.3%
Ethnicity African	99.8% (1241)	99.2–99.9%
Highest level of education:		
-Some primary	43.4% (538)	40.3–46.5%
-Some secondary	55.2% (687)	52.0–58.3%
-Some tertiary	1.4% (17)	0.7–2.4%
Religion:		
-Zionist Christian Church	41.0% (510)	38.1–44.1%
-Catholic	5.4% (67)	4.2–6.9%
-Protestant	52.7% (656)	49.6–55.8%
-Muslim	0.1% (1)	0.002–0.04%
Suicidal ideation	17.7% (220)	15.5–20.0%
Feeling depressed	67.8% (843)	64.8–70.6%
Sexually transmitted diseases	5.0% (62)	3.7–6.6%
Alcohol	10.3% (128)	8.6–12.4%
HIV–positive	12.0% (149)	8.7–16.2%
Smoking	2.0% (25)	1.3–3.0%

SE – standard error

*Some variation in numbers across variables due to a small amount of missing data.

**Table 2:** Factors associated with emotional abuse victimisation among girls in Swaziland, 2007 using bivariate logistic regressions (n = 1244)

Factor	Odds ratio (95% confidence Interval)	*P*–value
Physical abuse	2.55 (1.89–3.44)‡	0.001
Orphanhood	1.44 (1.09–1.90)†	0.009
Sexual abuse	2.11 (1.59–2.80)‡	0.001
Community trust (very low ref):		
-Low	0.70 (0.46–1.07)	0.103
-Undecided	0.61 (0.37–1.00)*	0.049
-High	0.73 (0.46–1.15)	0.180
-Very high	0.93 (0.47–1.83)	0.838
School trust (low trust ref):		
-Medium trust	0.89 (0.53–1.48)	0.640
-High trust	0.97 (0.60–1.58)	0.782
Religious practice	0.94 (0.80 –1.10)	0.451
Poverty	1.48 (1.12–1.96)*	0.005
Overcrowding	1.18 (0.78–1.78)	0.428
Close to mother	0.85 (0.77–0.92)‡	0.001
Close to father	0.94 (0.88–1.01)	0.119
Frequent caregiver changes	1.64 (1.21–2.22)‡	0.001
Age	0.97 (0.94–1.01)	0.147
Level of education primary (ref):		
-Some secondary	0.84 (0.63–1.12)	0.229
-Some tertiary	0.10 (0.01–0.78)*	0.028
Relationship status:		
-Married	1.31 (0.85–2.04)	0.225
-Living with partner, not married	1.17 (0.63–2.20)	0.614
-Not living with partner (ref)		
Urban or peri–urban area	0.72 (0.50–1.02)	0.066
Faith:		
-Catholic (Ref Zionist Christian)	0.56 (0.30–1.02)	0.062
-Protestant	0.83 (0.62–1.10)	0.197
-No religion	1.62 (0.42–6.27)	0.480
HIV–positive	0.96 (0.47–1.99)	0.918
Drinks alcohol	1.25 (0.79–1.96)	0.335
Smoking	0.91 (0.38–2.18)	0.839
Suicide ideation	2.23 (1.61–3.07)‡	0.001
Feeling depressed	2.15 (1.55–2.97)‡	0.001
Sexually transmitted disease	2.39 (1.29–4.41)†	0.006

*P < 0.05.

†*P* < 0.01.

‡*P* < 0.001.

**Table 3:** Multivariate logistic regressions of factors associated with emotional abuse among girls in Swaziland, 2007 (n = 1244)

	Model 1		Model 2		Model 3	
	Odds ratio	95% CI	Odds ratio	95% CI	Odds ratio	95% CI
Orphanhood	1.26	0.94–1.70				
Close to mother	0.91†	0.82–1.00	0.88†	0.80–0.89	0.88‡	0.80–0.97
Frequent caregiver changes	1.40†	1.00–1.97	1.41†	1.01–1.97	1.42†	1.03–1.97
Poverty	1.45†	1.07–1.96	1.52‡	1.13–2.05	1.51‡	1.12–2.03
Physical abuse	2.00§	1.45–2.75	1.98§	1.44–2.72	1.98§	1.45–2.71
Sexual abuse	2.19§	1.55–3.09	2.14§	1.52–2.01	2.22§	1.57–3.10
Tertiary education	0.17*	0.02–1.32	0.16	0.20–1.22		
Urban or peri–urban area	0.64*	0.43–0.96	0.64†	0.43–0.96	0.65†	0.44–0.96
Age	0.92§	0.88–0.97	0.92‡	0.88–0.97	0.92§	0.88–0.97
Faith	0.93	0.80–1.08				

CI – confidence interval

*Statistically significant at *P* < 0.1.

†Statistically significant at *P* < 0.05.

‡Statistically significant at *P* < 0.01.

§Statistically significant at *P* < 0.001.

**Table 4:** Logistic regression analyses of health factors associated with emotional abuse among girls in Swaziland, 2007 (n = 1244)

	Model 1		Model 2	
	Odds ratio	95% CI	Odds ratio	95% CI
Suicide ideation	1.79*	1.25–2.56	1.85*	1.30–2.63
Feeling depressed	1.87*	1.30–2.69	1.89*	1.31–2.71
Sexually transmitted disease	1.78	0.90–3.55		
Physical abuse	1.89*	1.38–2.58	1.92*	1.40–2.62
Sexual abuse	2.04*	1.45–2.86	2.08*	1.48–2.92
Urban or peri–urban area	0.78	0.53–1.16		
Age	0.88*	0.84–.93	0.89*	0.84–94
Faith	0.91	0.78–1.06		

CI – confidence interval

*Statistically significant at *P* < 0.05.

## RESULTS

1244 girls (mean age 17.9 years, 99.8% Black African) were interviewed in this study. Sample characteristics showed high levels of deprivation: 43.7% were orphaned, 58.5% experienced poverty to the point where there was insufficient food in the household, 26.1% experienced frequent caregiver changes and 87.8% lived in overcrowded accommodation. Of the participants, 67.8% reported feeling depressed, 17.7% suffered from suicidal ideation, 12.0% were HIV–positive and 5.0% had a sexually transmitted disease in the past (**Table 1**).

### Prevalence of emotional abuse in childhood

Nearly three in every ten participants (28.5%, 95% CI 25.8%–31.4%) reported experiencing emotional abuse at least once in their childhood. Of those who reported emotional abuse, 16.8% (95% CI 13.4%–21.0%) reported one incident, 24.8% (95% CI 20.7%–29.5%) reported few incidents and 58.3% (95% CI 53.2%–63.2%) reported many incidents. Girls in rural areas reported higher levels of emotional abuse compared to those in urban and peri–urban areas but this was not statistically significant.

### Emotionally abusive acts by family members compared to “others”

The majority of emotionally abusive acts were carried out by extended family members. The most frequent perpetrators were female relatives (27.8%, 95% CI 23.4%–32.6%), followed by male relatives (16.6%, 95% CI 13.0%–20.8%), biological mother (11.1%, 95% CI 8.45–14.5%), biological father (9.8%, 95% CI 7.0%–13.4%), brother (4.7%, 95% CI 2.9%–7.5%) and sister (4.3%, 95% CI 2.5%–7.4%). Less frequent perpetrators were ex–partners (1.2%, 95% CI 0.4%–3.5%), step–mothers (6.5%, 95% CI 4.5%–9.5%), teachers (2.5%, 95% CI 1.4%–4.6%) and community leaders (2.1%, 95% CI 1.2%–3.7%).

### Risk factors associated with emotional child abuse victimisation

Using bivariate regressions, childhood emotional abuse was associated with physical and sexual childhood abuse, orphanhood, poverty, and frequent caregiver changes. Being close to one’s mother was found to be protective against childhood emotional abuse as was being educated at tertiary level. Community trust, school trust, being close to one’s father, relationship status, overcrowding and religious practice were not associated with emotional abuse (**Table 2**).

Using multivariate regressions controlling for age, location, faith, frequent caregiver changes (OR 1.42, 95% CI 1.03–1.97), poverty (OR 1.51, 95% CI 1.12–2.03), childhood physical abuse (OR 1.98, 95% CI 1.45–2.71) and sexual abuse (OR 2.22, 95% CI 1.57–3.10) continued to be associated with an increased risk for childhood emotional abuse. Being close to one’s mother (OR 0.88, 95% CI 0.80–0.97) continued to be protective of emotional abuse. Orphanhood and tertiary–level education were no longer significant and therefore dropped from the model (**Table 3**, Model 3).

### Combinations of risk factors

For female youth in Swaziland, the predicted probability of childhood emotional abuse victimisation was 13.0% (95% CI 9.3%–15.6%) when none of the included risk factors of frequent caregiver changes, poverty, childhood physical and sexual abuse were present. One risk factor at a time was then included in a model, followed by a set of combinations of two, three and all four risk factors to establish which girls are at highest risk of emotional abuse.

Results for individual risk factors show the following predicted probabilities of emotional abuse expressed in percentages: 17.5% (95% CI 11.6%–23.3%) for poverty; 18.3% (95% CI 14.1%–22.4%) for frequent caregiver changes; 22.8% (95% CI 15.6%–30.0%) for physical child abuse; and 24.9% (95% CI–19.4%–30.0%) for sexual child abuse. When two risk factors were combined the predicted probabilities increased: 24.2% (95% CI 17.3%–31.1%) for frequent caregiver changes and poverty, 30.7% (95% CI 23.0%–38.5%) for child physical abuse and hunger, 29.5% (95% CI 20.2%–39.0%) for child physical abuse and frequent caregiver changes, 32% (95% CI 24.2%–40.0%) for child sexual abuse and frequent caregiver changes, 33.3% (95% CI 27.1%–39.5%) for child sexual abuse and poverty and 39.6% (95% CI 31.1%–48.1%) for child sexual and physical abuse. The combination of three risk factors showed an increase of predicted probabilities as well: 38.7% (95% CI 29.0%–48.5%) for physical abuse, frequent caregiver changes and poverty; 41.5% (95% CI 33.0%–50.0%) for sexual abuse, frequent caregiver changes and poverty; 48.3% (95% CI 38.8%–57.7%) for sexual abuse, physical abuse and frequent caregiver changes; and 49.7% (95% CI 41.5%–57.9%) for sexual abuse, physical abuse and poverty. Finally, among girls who reported experiencing all four risk factors, predicted probability for childhood emotional abuse victimisation increased to 58.4% (95% CI 49.8%–67.1%; **Figure 1**).

### Mental and sexual health outcomes associated with emotional abuse

Using *bivariate regressions*, childhood emotional abuse was associated with the following health risks: suicide ideation, feeling depressed and having suffered from a sexually transmitted disease. Being HIV–positive, consuming alcohol and smoking were not associated with emotional abuse (**Table 2**).

Using multivariate regression analyses, suicidal ideation (OR 1.85, 95% CI 1.30–2.63) and feeling depressed (OR 1.89, 95% CI 1.31–2.71) were associated with emotional childhood abuse when controlling for location, age, faith, physical abuse and sexual abuse. Having a sexually transmitted disease was no longer significant and was therefore dropped from the model (**Table 4**, Model 2).

## DISCUSSION

To the best of the authors’ knowledge, this is the first and only nationally representative study of emotional child abuse victimisation in Swaziland. This study adds to the literature on the epidemiology of emotional child abuse victimisation in sub–Saharan Africa by investigating prevalence, risk factors and health outcomes associated with emotional child abuse. It finds high prevalence of emotional abuse and significant associations with hypothesized risk factors and indicators of poor health. In addition, a cumulative effect of risk factors for emotional abuse could be established.

Prevalence of emotional child abuse was high, with 28.5% of girls reporting at least one incident and 58.3% of these reporting many incidents. Results also show that physical and sexual child abuse are strongly associated with emotional child abuse, suggesting many instances of multiple abuse victimisation. This finding corresponds to reports from other cross–sectional studies in sub–Saharan Africa showing associations between emotional and physical and sexual child abuse [8]. Poly–victimisation affects large numbers of children across the world [30]. Children affected by one type of violence are often susceptible to other types of violence as these are mediated by common individual, family and social vulnerabilities [31]. In a recent study from South Africa, 35.5% of children experienced more than five lifetime events of violence [32]. Factors associated with increased poly–victimisation in South Africa included parental substance use, parental absence due to illness, child substance use, child sexual risk behavior, single–parenting and living in urban areas [32].

Contrary to other research from the region [33], this study found female relatives to be the most common perpetrator of emotional abuse victimisation, followed by male relatives and biological parents. This may be due to family caregiving arrangements influenced by the high number of orphaned children in Swaziland, also found by this study. A large, national qualitative study on the drivers of violence affecting children in Swaziland, which included focus groups and interviews with 373 respondents, found that of all the interpersonal–level risk factors for violence against children suggested by respondents, family structure was the most cited. This is because “not living with biological parents” was perceived by many to almost guarantee negative differential treatment – which would be emotionally abusive at the least, but was also likely to result in a child being given a disproportionate number of household chores and harsher punishment [34]. While orphanhood was not associated with higher risk for emotional abuse in multivariate regressions, orphaned girls reported higher percentages of violence victimisation than non–orphans. This finding is consistent with a number of studies from sub–Saharan Africa [11,28,35,36]. However, these associations tend not to hold up in multivariate regressions controlling for other potential risk factors, which may drive risk for abuse more strongly than orphanhood alone. A recent systematic review examining risk for violence victimisation among orphaned and non–orphaned children found little or no difference in risk for abuse between the groups [37]. More research on the vulnerabilities of orphans is needed to better understand how orphanhood and violence interlink, particularly in countries with large numbers of orphans like Swaziland.

In line with high numbers of orphaned children, large numbers of girls reported frequent caregiver changes, and this type of social disadvantage is associated with emotional abuse. Conversely, being close to one’s mother was a protective factor. The association between frequent caregiver changes and emotional abuse in Swaziland is particularly interesting considering that relatives in the extended family but not biological parents were the most commonly reported perpetrators of emotional abuse. This pattern contrasts with some findings in South Africa [33]. Future research should investigate whether these relatives are the primary caregivers of the child and, if so, how the child came to be in their care (ie, through orphanhood, abandonment, work or migration) as well as whether these abusive events occurred while the child was in the primary care of her biological parents or outside the household setting. Caregiving arrangements for girls should also be investigated to determine whether having kinship caregivers as opposed to non–kinship carers increases the odds of emotional abuse victimisation as has been found in qualitative research in Swaziland [34].

Overall, the findings correspond with research from South Africa where multiple victimisation, poverty and frequent caregiver changes were found to be associated with emotional abuse [11]. It is also likely that the specific context of Swaziland compounds these problems. Swaziland has a population of 1.3 million, 59.1% of whom live on less than US$ 2 per day [38] and an estimated population of 120 000 orphaned children [39]. The HIV prevalence in Swaziland is 27.4%, which is the highest prevalence worldwide [40]. It is therefore likely that families experience multiple severe stressors such as extreme poverty, illness and death, resulting in insufficient care, disruption of social norms, and overburdened and under–resourced systems for child protection [14,41-43]. Violence prevention interventions could thus be linked to HIV–prevention efforts through comprehensive social protection and social welfare efforts [44-46].

Contrary to other studies, overcrowding was not associated with emotional abuse in the multivariate analyses [8], which may be a result of the way in which this variable was measured. Establishing the highest number of residents in the household throughout one’s lifetime may not be sensitive enough to establish whether the overcrowding was short–term or long–term.

Cumulative effects of risk factors on emotional abuse victimisation were found. Without any of the three significant risk factors, a girl’s probability of experiencing childhood emotional abuse was 13%, while girls who reported frequent moves, hunger, and physical and sexual childhood abuse had a probability of 58.4%. The connections between adverse childhood experiences such as orphanhood, parental mental illness or childhood abuse, and putative outcomes have long been established in high income countries [47] with a growing evidence–base in low– and middle–income countries [48,49]. In particular, adults who experienced a combination of four or more adverse events in childhood are at risk for negative health outcomes.

Feelings of depression and suicidal ideation were also found to be associated with emotional abuse in this study. While more robust measures on mental health would need to be used in future studies to explore this relationship, this association has also been found in the evidence from the sub–Saharan African region [5] and across the globe [50]. Emotional childhood abuse may contribute to the salience of suicide as a leading cause of death for young people in southern Africa [51]. Sub–Saharan Africa experiences a high burden of child and adolescent mental health problems with a recent systematic review of community–based studies establishing a psychopathology prevalence rate of 14.3% [52] but with little nationally representative data available from the region [53].

The results of this study suggest a need for comprehensive child abuse prevention and protection strategies for girls in Swaziland. Girls experiencing multiple adverse conditions are at high risk of abuse, and humiliating punishment of children is an accepted form of discipline in many Swazi households [17]. Holistic social support interventions that promote positive parenting behaviors of primary caregivers may be useful [54] and help to build stronger relationships between children and their caretakers, particularly mothers. A recent study from South Africa found adolescent health risks were linked to family disadvantage via abusive parenting and caregiver mental health problems, suggesting a need for combined parenting assistance, poverty alleviation and mental health support [10]. In particular, adolescent mothers are at high risk for harsh parenting [55], struggle with parenting stress [56], and experience high levels of poverty [56,57] and low levels of education [58]. Considering the high risk for re–victimisation in victims of childhood violence [50,59] and the possibility of inter–generational violence transmission to these girls’ children [60], the high prevalence of abuse victimisation in this sample suggests that an entire society is persistently at risk for poor outcomes. Programmes are thus needed for both adult and adolescent mothers to reduce household stress and increase parenting capacity. Emerging evidence on parenting programmes with financial strengthening components for caregivers and teenagers shows clear reductions in physical and emotional abuse victimisation [61], also in the context of high levels of caregiver changes [62] in South Africa.

In light of the high levels of physical and sexual violence found in this study, a holistic approach to prevention is needed. As described in the INSPIRES framework, the seven strategies with the best available evidence for the reduction and prevention of violence against children across contexts are implementation of enforcement laws, changing of norms and values, safe environments, parent and caregiver support, income and economic strengthening, response and support services, and education and life skills, which should be implemented as part of a comprehensive and multi–sectoral plan [63]. However, each of these strategies need to be adapted and evaluated for the contexts in which there are to be used. Emerging research from other sub–Saharan countries suggests that prevention of violence against girls should incorporate asset–based approaches. Protective assets can be income generating activity, friendship networks, specific knowledge of one’s community, services and rights, enabling girls to make safety plans, and creating safe spaces [64]. In Swaziland specifically, a school–based intervention of “Safer Spaces” showed stark increases in girls’ protective assets and in knowledge about gender–based violence. Furthermore, changes in gendered attitudes were observed, which is often the first stage in normative transformations around violence. However, reporting of violence increased and no reductions in victimisation were shown [65]. Early results from other countries in sub–Saharan Africa show a potential for using protective assets to prevent gender–based violence [64], early child marriage [66] and HIV–risk [67]. However, further research and large–scale intervention evaluation are needed in order to verify the impact of parenting interventions and protective assets on child abuse prevention.

Research in sub–Saharan Africa has consistently shown the impact of violence on educational attainment [68,69] as well as the protective effect of secondary education on harsh parenting in adulthood [70] and exposure to intimate partner violence [71]. A case could thus be made that secondary schooling – provided the school setting is safe and protective as indicated in the Safe Spaces Intervention – is another important child abuse prevention intervention.

In order to address the multiple needs of young women and children in Swaziland, an integrated, early intervention approach may be effective. This holistic approach could give ample opportunity for different services providers to collaborate and address multiple forms of violence using gender–sensitive approaches. Where strong links are built between services for women and children as well as between school–based and parenting programmes, complex family dynamics may be addressed, and continued programming for adolescent health and development with gender–sensitive content could be facilitated.

### Limitations and future research

This study had a number of limitations. First, an all–female sample was recruited so no assumptions regarding the victimisation of boys can be made. Second, data were cross–sectional and therefore did not allow for causal inferences. Further, it is impossible to say in what temporal order risk factors and putative health outcomes occurred. In other words, we cannot know if children experience suicidal ideation because they were victims of violence or if they are more vulnerable to experience violence. Third, the study used retrospective self–report which may be subject to recall bias due to under–reporting of abusive events [72]. Furthermore, the study used interviewer–guided questionnaires, which may have resulted in some under–reporting due to social desirability bias in particular with regards to sensitive questions such as parental aggression and sexual violence. However, official records are not kept reliably in Swaziland, and few child abuse victims access services [73], thus making self–report a more reliable source of information. Further, the study did not use internationally validated measures of child abuse victimisation and mental health. For example, emotional abuse was measured using one item that grouped many emotionally abusive acts in one question. While different abuse measures use differing definitions of emotional abuse, there is large overlap between the abusive incidents queried in the single–item question and the most common child abuse measures. However, including four types of abusive behavior in a single question might have made this question too complex and thus led to over– or under–reporting of abusive events. Depression was measured with only one question about whether the participant had felt depressed. Validity of these single items as indicators of complex constructs is not established. Although these single–item measures have been used in multiple VAC studies in sub–Saharan Africa and elsewhere [27] since the completion of this study and the basic trends in prevalence may be similar to regional surveys that used more comprehensive measures, ie, Optimus Study in South Africa [74], future research should develop and utilize validated and standardised measures. Finally, there is a strong likelihood for unmeasured confounding in this study. Even though models adjusted for potential confounding, no such adjustment was possible for caregiver–related variables commonly associated with emotional abuse victimisation (ie, mental health problems, drug abuse) or community factors (ie, social norms, service availability, girls’ knowledge of services).

## CONCLUSIONS

Overall, the findings of this study demonstrate the magnitude of girls’ exposure to emotional abuse in Swaziland. It highlights particularly vulnerable groups such as victims of multiple types of abuse, those who experienced frequent caregiver changes and those most affected by poverty. The findings are relevant to social policy and intervention programmes. Holistic child abuse prevention programmes targeting poverty, unstable family environments and parenting—such as cash transfers and programmes focused on keeping girls in education, teaching parenting skills and delaying marriage—are needed to reduce the burden of emotional abuse and associated adverse mental health outcomes.
